# Navigating disclosure in new romantic partnerships as an adolescent or young adult with Li-Fraumeni syndrome

**DOI:** 10.1007/s10689-025-00495-3

**Published:** 2025-09-10

**Authors:** Camella J. Rising, Chloe O. Huelsnitz, Rowan Forbes Shepherd, Catherine Wilsnack, Patrick Boyd, Alix G. Sleight, Ashley S. Thompson, Sadie P. Hutson, Payal P. Khincha, Allison Werner-Lin

**Affiliations:** 1 Clinical Genetics Branch, Division of Cancer Epidemiology and Genetics, National Cancer Institute, 9609 Medical Center Drive, Rockville, MD 20850 USA; 2 Behavioral Research Program, Division of Cancer Control and Population Sciences, National Cancer Institute, Rockville, USA; 3 Department of Population Sciences, City of Hope, Duarte, USA; 4 Department of Occupational Science and Occupational Therapy, University of Southern California, Los Angeles, USA; 5 Division of Nursing, The University of Texas MD Anderson Cancer Center, Houston, USA; 6 School of Social Policy and Practice, University of Pennsylvania, Philadelphia, USA

**Keywords:** Adolescents and young adults, Li-Fraumeni syndrome, Genetic, Cancer, Relationships, Communication, Disclosure

## Abstract

Li-Fraumeni syndrome (LFS) is an early-onset cancer syndrome caused by pathogenic germline *TP53* variants. Adolescents and young adults (AYAs) with LFS may have challenges navigating new romantic partnerships given the significant effects of LFS on multiple life domains that also affect partners (e.g., reproductive decision-making). Disclosing LFS-related information to new partners may be especially difficult given the uncertainty, complexity, and chronicity of LFS. This qualitative-descriptive study aimed to explore AYAs’ LFS disclosure decisions and experiences in new romantic partnerships. Participants were individuals with LFS aged 15–39 years at enrollment in a National Cancer Institute study. The analytic sample included 33 AYAs who completed at least one telephone interview. Greene’s disclosure decision-making model guided thematic analysis. Participants were mostly female (67%) and married/in a long-term relationship (58%), with mean age 29 years and ≥ 1 primary cancer (61%). Key factors in LFS disclosure decision-making included perceived relevancy to partners (e.g., future children’s genetic risk), partner traits (e.g., trustworthiness), and relationship quality (e.g., closeness). AYAs described LFS disclosures in new partnerships as a process. Disclosing LFS diagnosis often occurred early to fulfill a sense of moral obligation and emotionally self-protect from future rejection, while subsequent LFS disclosures depended on relationship quality and the topic’s emotional valence or complexity. Partners often earned AYAs’ trust by demonstrating a willingness to learn about and try to understand LFS. Clinicians and LFS communities could support AYAs by providing opportunities to discuss, normalize, and ameliorate challenges with LFS disclosures in new romantic partnerships (e.g., peer support groups, psychotherapy).

**Supplementary Information:**

The online version contains supplementary material available at 10.1007/s10689-025-00495-3.

## Introduction

Adolescents and young adults (AYAs) with heritable cancer syndromes, such as Li-Fraumeni syndrome (LFS), hereditary breast and ovarian cancer (HBOC) syndrome, and von Hippel-Lindau disease (VHL), face numerous psychosocial challenges during critical developmental periods [1, 2, 3, 4]. Intensive cancer screenings, risk-reducing procedures (e.g., mastectomy), cancer treatments, and family cancer diagnoses and bereavement are among several factors that may cause significant disruptions in psychological, emotional, and social health and well-being. These challenges may also disrupt AYAs’ pursuit of developmentally normative social goals, such as forming romantic relationships [4, 5, 6].

Self-disclosure, the process of revealing personal information about oneself to others, is key to the formation and development of personal relationships [7, 8] yet disclosing and discussing the consequences of a heritable cancer syndrome [4, 5] or an early-onset cancer diagnosis [9, 10] with new romantic partners can be difficult for some AYAs. Self-disclosure has inherent social risks (e.g., rejection), but also social rewards (e.g., acceptance, closeness). Findings from HBOC and VHL research have demonstrated that young adults often fear rejection after disclosing their diagnosis to new partners due to factors such as feeling less desirable, reproductive risks, and partners’ perceptions of syndrome severity or effects on the body (e.g., loss of breasts, scars) [4, 5, 6]. However, young adults also experience relational benefits after disclosing a cancer syndrome diagnosis, such as deepening trust [4, 5].

In the case of LFS, less is understood about how AYAs, individuals aged 15 to 39 years [11], navigate self-disclosure in new romantic relationships. LFS is an early-onset autosomal dominant condition primarily caused by pathogenic/likely pathogenic germline *TP53* variants, conferring nearly 100% lifetime cancer risk from birth and a 50% chance of passing the germline variant to each biological child. Individuals with LFS are at risk of multiple primary cancers and a shortened life expectancy [12, 13]. Median age at first cancer diagnosis is 33 years in women and 45 years in men [12]. LFS risk management involves intensive multi-modal cancer screenings to detect cancers at early stages [12, 14]. Currently, the only option for primary cancer prevention is risk-reducing mastectomy for females [15, 16].

**Fig. 1:**
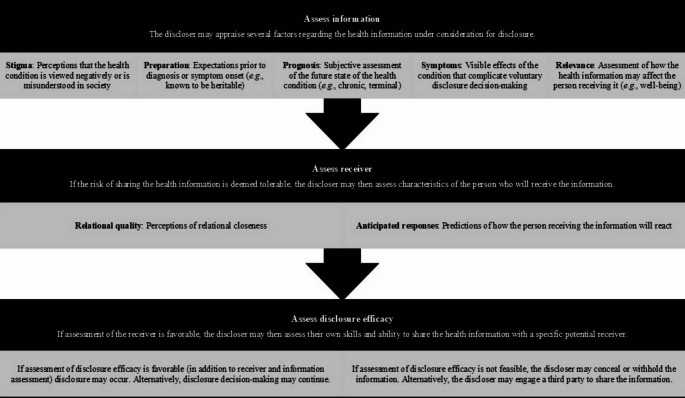
Disclosure decision-making model concepts and definitions [14]

Greene’s disclosure decision-making model (DD-MM) [17, 18], previously applied in non-LFS cancer syndrome contexts (e.g., *BRCA1/2* [19]), is a theoretical framework that may offer insights into how aspects of LFS make self-disclosure to new romantic partners especially difficult for AYAs. The DD-MM considers multiple factors that individuals assess before choosing to share or withhold health information from another individual (e.g., diagnoses, health updates). According to the DD-MM, disclosure decision-making is informed by features of the health condition (*assess information*), partner appraisals (*assess receiver*), and perceived ability, confidence, and skill to disclose information about the condition (*assess disclosure efficacy*). Figure 1 further delineates key DD-MM constructs and definitions [17, 18, 19]. Factors that may influence LFS disclosure decision-making include substantial illness uncertainty, cancer survivorship issues, and genetic risk to future biological children, all potentially relevant to romantic partners. Moreover, some AYAs may lack experience, confidence, or skills communicating about high genetic cancer risk [4, 5].

While prior research in LFS [20, 21] and non-LFS cancer contexts [4, 5, 6, 22, 23, 24] has investigated AYAs’ experiences navigating disclosure and dynamics in social relationships (e.g., friendships, family relationships, romantic partnerships), past studies have not investigated how LFS influences the dynamics and functioning of AYAs’ *new* romantic partnerships after disclosing an LFS diagnosis. Compared with other types of close relationships, romantic partnerships often involve higher mutual influence between partners, shared environments (e.g., cohabitation), and interwoven lifestyles (e.g., finances, childcare responsibilities). In the context of chronic health conditions, such as cancer, romantic partners are often informal caregivers [25]. Thus, although LFS can have lasting and far-reaching implications for all types of close relationships, its consequences are especially significant for the formation and development of romantic partnerships.

In forming new romantic partnerships (i.e., dating), partners exchange personal information on a range of topics that affect how the relationship develops. Disclosure of an LFS diagnosis not only provides important information to the partner without LFS but also provides an opportunity for the individual with LFS to observe how their partner reacts to the disclosure. Past studies of populations living with heritable cancer syndromes [26], including couples predominantly in middle adulthood living with LFS [21], indicate that the way individual partners and couples communicate about LFS (e.g., restrained vs. open communication) can influence relational outcomes (e.g., intimacy, trust). Given the young age at which LFS-related cancers and LFS diagnosis may occur, AYAs may need support to disclose LFS and cultivate adaptive LFS communication behaviors in the context of new romantic partnerships, yet their support needs remain largely unexplored in research.

This qualitative-descriptive study aimed to address these research gaps by exploring the experiences, perspectives, and potential support needs of AYAs regarding LFS disclosure decision-making in the context of new romantic partnerships. To better understand motivations, challenges, and outcomes of LFS disclosure to new partners, we posed the following research questions: (RQ1) What aspects of LFS and the new romantic partner(ship) do AYAs appraise when making LFS disclosure decisions? (RQ2) How confident and skillful do AYAs feel self-disclosing LFS? (RQ3) How do AYAs approach LFS disclosure (i.e., message strategies)? (RQ4) What are common outcomes of AYAs’ LFS disclosure?

## Methods

This study was approved by the National Institutes of Health (NIH) IRB under the National Cancer Institute (NCI) LFS study (NIH Protocol 11-C-0255, ClinicalTrials.gov, Identifier NCT01443468). Eligibility criteria included diagnosis of a pathogenic/likely pathogenic germline *TP53* variant and being aged 15 to 39 years at study recruitment. Analysis of qualitative data was nested in a larger longitudinal mixed methods study of AYAs’ experiences living with LFS (methods for this study have also been described elsewhere [27, 28]). Two waves of qualitative data collection occurred between October 2019 and July 2021. Serial interviews were conducted to enable assessment of long-term psychosocial sequelae of LFS during a time of rapid developmental change (i.e., the AYA lifespan phase). All participants provided consent to participate in this study; parents provided consent for minors.

Specific to new romantic partnerships, the wave 1 interview guide included questions regarding participants’ experiences with LFS communication and social support in family and other social networks. Wave 1 data informed development of the wave 2 interview guide, which elicited depth regarding LFS-related interactions in dating and new romantic relationships, specifically (Online Appendix 1). Four qualitatively trained NCI researchers conducted audio-recorded telephone interviews. The average interview length was about 70 min for wave 1 interviews (*n* = 38) and about 80 min for wave 2 interviews (*n* = 30). The average time between interviews was 16 months (range = 13–21 months).

We thematically analyzed combined data from wave 1 and 2 interviews [29]. Each interview was treated as its own case (i.e., interviews completed by one person were analyzed separately). A seven-person coding team developed an initial codebook for wave 1 by applying an inductive-deductive open coding approach. The coding team discussed and resolved coding discrepancies over the course of several meetings to finalize the codebook with code definitions and usage parameters. To develop the initial wave 2 codebook, coders utilized the same inductive-deductive open coding approach and applied codes from the final wave 1 codebook where relevant.

A subset of coders then conducted a second level of analysis. Deductive coding in this phase of analysis involved applying a priori codes derived from the DD-MM [17] and relevant thematic findings from relationship-focused research that included AYAs with non-LFS heritable cancer syndromes [4, 5] and a sample of primarily middle-aged adults with LFS and their partners [21]. Deductive codes from these sources were developed directly from published constructs, themes, and respective definitions (e.g., DD-MM constructs, Fig. 1) and then adapted, where needed, for the developmental and clinical context of AYAs with LFS. Of the 42 total participants interviewed, 33 provided data relevant to current or past experiences disclosing LFS to new romantic partners. Thematic analysis involved assigning codes to text in Dedoose version 9.0.107, grouping codes by thematic salience [30], and identifying properties of each theme [31]. Strategies to ensure analytic rigor included memo writing, discussion, and verification of final themes with the coding team [32].

## Results

### Participants

Table 1 describes characteristics of the analytic sample (full sample characteristics are described elsewhere [27]). Mean age was 29 years, and most were female (67%) versus male (33%). The majority had a history of one or more primary cancers (61%), and most of these individuals were female (85%). Mean age was 22 years at first primary cancer diagnosis and 23 years at genetic diagnosis. More than half of females had a history of mastectomy (59%). The majority reported being married or in a romantic partnership for more than five years (58%) and did not have children (61%).

**Table 1:** Demographics

Characteristic			Analytic sample (*n* = 33)
*Age (years)*			
Mean (SD)			29 (6.2)
Range			17–41
*Age at first primary cancer diagnosis (years)*			
Mean (SD)			22 (9.4)
Range (years)			0.5–35
*Age at genetic testing (years)* ^a^			
Mean (SD)			23 (6.9)
Range			2–35
*Biological sex* (*n*,* %)*			
Male			11 (33.3)
Female			22 (66.7)
*Race (* *n, %)*			
White			29 (87.9)
Multiracial			3 (9.1)
Unknown			1 (3.0)
*Ethnicity (* *n, %)*			
Hispanic/Latino			2 (6.1)
Non-Hispanic/Latino			31 (93.9)
*Educational attainment*^b^ (*n, %)*			
Attending high school			1 (3.0)
Attending college			1 (3.0)
Non-college graduate			9 (27.3)
College graduate			14 (42.4)
Unknown			8 (24.3)
*Cancer status*^c^ (*n, %)*			
No cancer			13 (39.4)^d^
1 primary cancer			14 (42.4)^e^
≥ 2 primary cancers			6 (18.2)^f^

^a^Unknown for 2 participants

^b^Non-college graduates include those whose highest educational attainment is a high school diploma/GED or post-high school training (technical or vocational school). College graduates include those whose highest educational attainment is an associate, bachelor’s, or graduate degree

^c^The most frequently diagnosed cancers were female breast (*n* = 11) and brain (*n* = 7). Other cancers included liver, thyroid, adrenal cortical carcinoma, and soft tissue sarcoma

^d^Included 5 females and 8 males

^e^Included 12 females and 2 males

^f^Included 5 females and 1 male

## Disclosure in new romantic partnerships: experiences, perspectives, and challenges

Two major themes identified in combined data from wave 1 and 2 interviews included *LFS disclosure decision-making* and *LFS disclosure outcomes*. Guided by the DD-MM [17], we grouped three subthemes under *LFS disclosure decision-making*: *assessing the diagnosis*, *assessing the receiver*, and *assessing disclosure efficacy*. Two subthemes were grouped under *LFS disclosure outcomes*: *opening or restraining subsequent LFS disclosures* and *learning LFS*. In text, illustrative quotes are accompanied by participants’ respective pseudonym, age range in years corresponding to developmental phase, and cancer history (*no cancer* vs. *cancer survivor*). Developmental phases were defined as late adolescence (aged 15–17 years), emerging adulthood (aged 18–29 years), and early adulthood (aged 30–39 years) [33, 34].

## LFS disclosure decision-making

…before we get any deeper than just saying boyfriend and girlfriend, I should probably let you know that I have a rare genetic disorder. (Chelsea, 18–29, cancer survivor)

Nearly all AYAs had experience disclosing their LFS diagnosis to a new romantic partner and described a thoughtful and strategic LFS disclosure decision-making process that involved assessing several aspects of LFS, the receiver, and disclosure efficacy. Together, these appraisals influenced whether, when, and what AYAs shared specifically about their LFS diagnosis. Although sharing the information often felt “scary,” a sense of moral obligation strongly motivated relatively early disclosure to new partners, some describing it as the “right thing to do” out of fairness, honesty, and respect for their partner. Disclosing LFS and LFS-related cancers early in the relationship also served as an emotional self-protection strategy for some AYAs who reasoned that if partners responded by rejecting them, they would rather it happen when they were less emotionally invested.

### Assessing the diagnosis

AYAs’ disclosure decision-making process was strongly affected by the anticipated impact of LFS on partners. Veronica (18–29, no cancer) said, “It’s like a big bomb on the other person because there is such a high percentage [likelihood] of your significant other getting cancer.” Several AYAs reported that they had disclosed their LFS diagnosis early in relationships because (potential) cancers might affect partners emotionally and practically, including taking on a caregiving role. To protect partners’ future well-being, they felt a moral responsibility to be transparent about the seriousness of LFS as a health condition. Disclosing LFS also fulfilled a sense of moral responsibility to inform partners about the genetic risk for future biological children and provide information that might facilitate any future discussions about parenthood. Carrie (30–39, cancer survivor) shared that telling her boyfriend, now husband, early in their relationship “was the responsible and right thing to do,” especially because they were making enduring life choices:

He had to be okay with [my decision about] never having biological kids… He had to be okay knowing there could potentially be a time where he was going to have to take care of me…I didn’t feel it was fair to be in a relationship with somebody when they didn’t know that part.

George (30–39, cancer survivor) described similar considerations, but added that the potential life-limiting nature of LFS would also influence his decision to disclose to a future partner:

That person is going to need to know my situation and what that possibly means being in a relationship with them and what that could mean if kids are a thing down the road. And not only that, just the potential that I could get cancer and be dead any time from the next couple years to 10 to 15 years down the road. How is that going to affect them?

Despite most viewing disclosure of LFS as morally important, some AYAs recounted moments of hesitation in revealing their LFS diagnosis to a new partner due to ongoing physical effects of cancers, treatments, risk-reducing procedures, or perceived stigma. In sharing the challenging effects on her body and self-identity, Marley (18–29, cancer survivor, post-mastectomy) offered deeper, underlying explanations regarding why some AYAs may avoid LFS disclosure, dating, or long-term romantic attachments:

As a woman… I feel like now that I’m not able to have kids and I don’t have my boobs, it just kind of turns me into a half woman. Not that I don’t feel like I’m worth somebody’s time… but it definitely makes it hard to be in relationships and explain what happened to you. And sometimes, you don’t even feel like explaining.

Acute events that required medical attention or intervention, such as an abnormal cancer screening result, a new cancer diagnosis, or a post-mastectomy complication, necessitated earlier disclosure to new partners than planned. Lauren (30–39, cancer survivor) shared that she disclosed her LFS diagnosis to a dating partner of one month because one of her post-mastectomy breast implants ruptured, requiring surgery. She recalled apologizing to him, “Hey, sorry, I’m going to be off the radar for a little bit. I’m going to be healing and whatnot.” Silver (30–39, cancer survivor) had even less time to prepare a disclosure strategy to a new partner due to a new cancer diagnosis, acute symptoms, and impending treatment:

I found out that I might have a brain tumor… [It] was one week after we met… I waited a few weeks until we knew we were going to do something about [the tumor] because I thought at first it was going to be like a wait and watch situation. But then I started having some focal seizures and [my doctors] were like, “No, we need to start pursuing treatment.” That was when I told him that I had Li-Fraumeni also.

### Assessing the receiver

Perceptions of partner traits and relationship quality strongly influenced AYAs’ final decision on whether and precisely when and what LFS-related information they would share. Based on prior dating experiences, Silver developed heuristics around when she would disclose her LFS-related cancer diagnosis to new partners:

As I’ve been dating, I’ve often taken the strategy of telling people about having had breast cancer relatively early on. Sort of on something between the first and fourth date, depending on when it feels right, or it comes up… Also, if a date’s just going really badly…I just don’t tell them because… why would I bother emotionally opening up about this?

AYAs shared that trust, understanding, respect, and supportiveness were high on their partner “checklist” before disclosing their LFS diagnosis. Veronica described her thought process before disclosing to her boyfriend:

It’s the level of trust… So, I had a checklist in my brain. Of like, okay, I can trust him. He’s very understanding. I believe he’ll be very supportive and very respectful of my wishes and my process [related to LFS] …And making sure it’s a safe place and relationship for me to tell him all that because it is such a huge thing in my life.

Anticipating negative responses, some AYAs described LFS self-disclosure as “scary,” especially when they could envision themselves with a partner long term. Jackie (30–39, cancer survivor) recalled how afraid and nervous she was to broach the subject of her LFS with her then boyfriend, now husband:

I was really, really scared to tell him that I had LFS because I could tell he was falling in love with me and I was basically about to tell him, “I know you’re really starting to fall for me, but just so you know, I’m probably going to die of cancer before we’re old and gray.”

Also anticipating rejection, Cindy (30–39, cancer survivor) delayed disclosing her LFS and cancer history when she first started dating her now husband:

I was really hesitant to tell him about the cancer and the LFS. So, I tried to stay away from him… I tried to avoid any communication with him. He kept calling me… Finally, I tried to just tell him the truth and asked what he thought.

Eventually, her husband’s disclosure of his own chronic illness helped Cindy share her LFS diagnosis. Sharing their health stories enabled the couple to mutually support each other through their respective health challenges early in the relationship, as Cindy went on to say:

He had some health issues as well, and I didn’t know at that time. So, we both had secrets from each other… We chose to disclose and tell the other one what we had. In that way, we could really support each other.

### Assessing disclosure efficacy

Some AYAs mentioned not feeling confident or skillful sharing information about their LFS diagnosis with new romantic partners. Silver (30–39, cancer survivor) felt LFS disclosure was a major issue facing AYAs with LFS and emphasized, “It’s a hard thing to talk about in dating and *how* to tell a new partner that.” Low disclosure efficacy was especially evident among participants who had never experienced disclosing to a partner. Jenny (18–29, no cancer) recognized the importance of being open and honest about her LFS diagnosis, but she felt anxious even thinking about telling a future partner:

I’m worried I don’t have the proper information [about LFS] or I won’t be able to articulate my thoughts properly to my significant other when the time comes… I’m still very confused. I’m not great at communicating…A lot of [anxiety] is around communicating thoughts and feelings, and [LFS] diagnosis and information… I do not know if what I’m saying is 100% accurate.

Ishmael (18–29, no cancer) shared that disclosing his LFS diagnosis to a future partner “would be big.” He said: “A tough part for me is deciding when to tell or how to tell my significant other. Whether that’s early in the relationship or it’s later… I mean it’d be a tough conversation to have, for sure.”

## LFS disclosure outcomes

Oftentimes, and to many AYAs’ surprise, new romantic partners’ reactions to disclosure of LFS or an LFS-related cancer diagnosis were relatively “uneventful” and supportive, allaying fears around sharing the information. Jackie mentioned feeling relieved by her partner’s reaction: “When I told him [about having LFS] … he was like, ‘Oh, my goodness. Is that all? Is that all?’…He took it so well and so supportively.”

Several AYAs reported that if new relationships progressed after disclosing their LFS diagnosis, they faced difficult decisions about determining what additional LFS information to share with partners (e.g., life-limiting nature of LFS, reproductive beliefs). As assessed for the initial disclosure of LFS diagnosis, subsequent disclosures involved assessing features of the LFS information, the receiver, and disclosure efficacy. Subsequent LFS disclosures, perceived responsiveness of partners (e.g., asking questions, listening, expressing support), closeness, and trust appeared to be strongly interrelated.

### Opening or restraining subsequent LFS disclosures

Following disclosure of an LFS diagnosis, decisions about what and when to provide new partners with additional LFS information varied among AYAs. Several described LFS disclosure in the context of new romantic relationships as a process rather than a single event. Believing she had an important role in helping her boyfriend learn about the complex nature of LFS (e.g., high genetic cancer risk, cancer screening recommendations, primary prevention options), Veronica offered to answer his questions any time. His responsiveness resulted in her feeling emotionally supported:

He asked a lot when I first told him [about my LFS diagnosis] … [W]e’re… going on five months now, [and] he’s still asking questions every so often… He’s trying to better understand what my life is like and…[what] he can do to help me and how he can support me the best he can.

George, in contrast, decided to withhold additional LFS information (e.g., cancer screening appointments, emotional effects of LFS, medical decision-making) from his then-girlfriend. Her seeming lack of interest in understanding LFS after he disclosed his LFS diagnosis led to silence about his experiences and challenges, which he felt he had to cope with alone:

I wanted her to be a part of [LFS] conversations…[A]fter a certain point, I stopped bringing that information into our conversations, and I just kept it to myself. She never really asked about it…A very, very big factor going forward [is] someone that…wants to be there when I hear shitty [LFS-related] news and wants to have conversations about how that makes me feel and what I’m going to do about it….

For some AYAs, the LFS disclosure process involved waiting to broach certain LFS topics, such as the life-limiting nature of LFS or reproductive beliefs. Prominent reasons for withholding complex, emotion- or morally-laden LFS information included not feeling emotionally ready (personally or in the relationship) and not wanting to overwhelm new partners. Allan (18–29, no cancer) shared that he felt emotionally unprepared for the “I might not be around as long as you are conversation,” as he put it. Allan’s fears about his girlfriend’s possible rejection highlight that LFS-related disclosures may continue to feel scary for AYAs well beyond initial disclosure of LFS diagnosis. He said:

Some of the conversations that we haven’t really had are along the lines of, would you want to be in a long-term, married relationship to somebody that potentially has a shorter life expectancy?… I fear that she says, “I don’t think I want to go on and be in a relationship with someone with LFS.”

### Learning LFS

Several AYAs mentioned that new romantic partners’ willingness to learn about and try to understand LFS early in the relationship was a strong sign of caring and support. Chelsea said about partners: “They need to understand exactly what happens with LFS, which is why with my current boyfriend, I’m so excited he wants to learn about LFS and everything that comes with it.” Partners tended to learn about LFS through open communication with AYAs, attending medical appointments with them, or by experiencing or witnessing LFS consequences, such as the burdens of cancer screening, AYAs’ cancer diagnoses and treatments, or the LFS-related deaths of AYAs’ family members.

AYAs often interpreted a partner’s willingness to remain in the relationship after learning about and more deeply understanding the consequences of LFS as a sign of trustworthiness. AYAs felt they had a strong relationship when partners proved themselves “to be there no matter what.” Karen (18–29, cancer survivor), who was married at the time of her LFS diagnosis, shared about her husband:

Seeing that he didn’t disappear or get scared and run away is [a] testament to the person I know him as… [The LFS diagnosis] really didn’t faze him. And like, oh wow, you’re too much, you know? Between the cancer and the LFS.

Chelsea underscored the importance of a partner’s willingness to remain in the relationship despite LFS-related challenges they may face as a couple. She shared about a past boyfriend who did not “stick around:”

I think the longer he was involved…the more he came to grips with [LFS] and the more it… I don’t know if intimidated is the word…But it’s kind of easy to tell people what it is and for people to say, “Oh, that’s perfectly fine” [after disclosing an LFS diagnosis]. But the test comes in when they actually stick around, when you have health things go on like a preventative surgery or another cancer diagnosis…It’s [about] who sticks with you during the hard times.

**Fig. 2:**
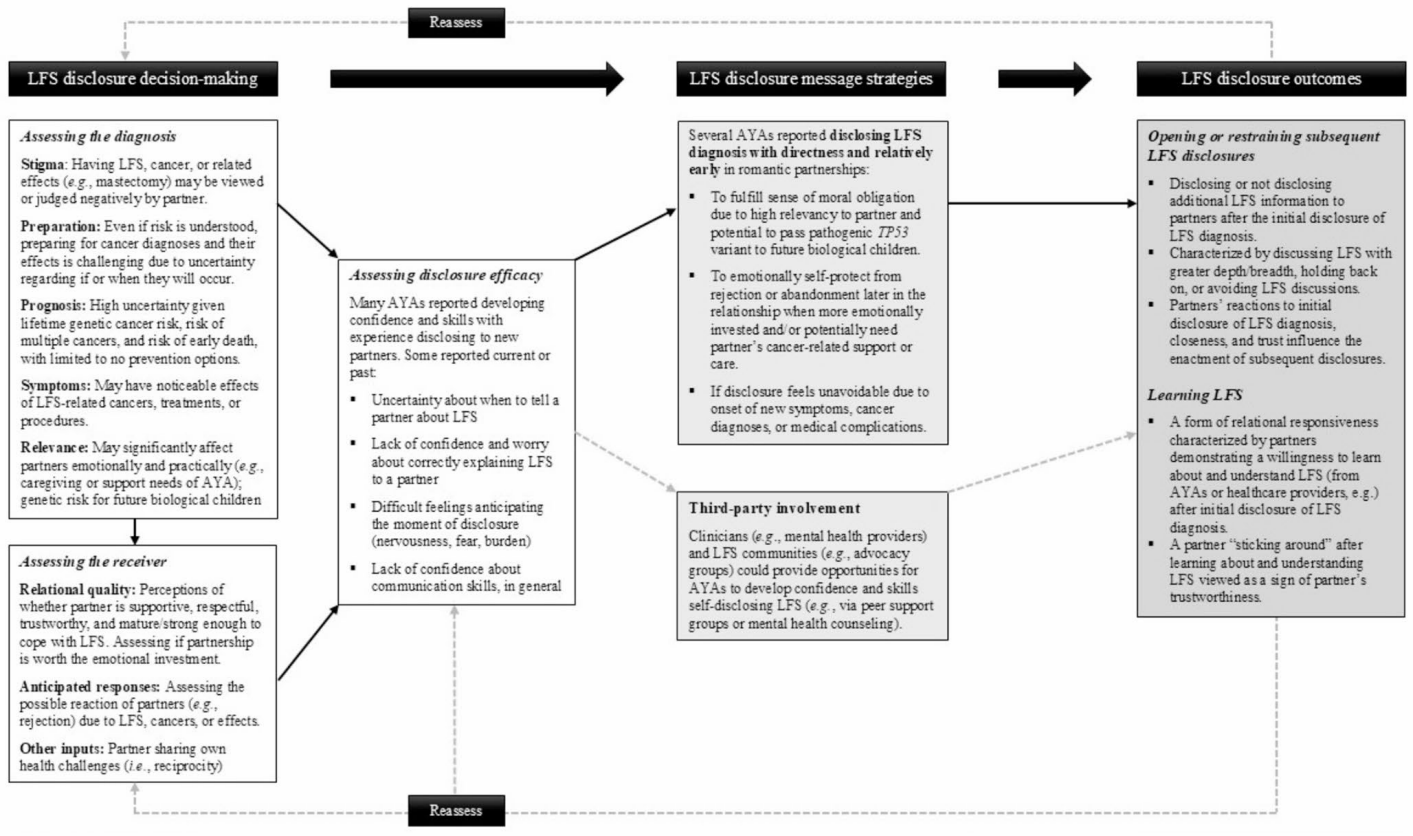
AYAs’ LFS disclosure experiences with new romantic partners

Figure 2 uses the DD-MM to illustrate AYAs’ LFS disclosure experiences with new romantic partners.

## Discussion

### Principal findings

Participants reported that many aspects of LFS, new romantic partner attributes, and relationship quality affected their LFS disclosure decisions in accordance with Greene’s disclosure decision-making model (DD-MM) [17]. The significant effects LFS could have on partners’ lives strongly motivated disclosure; however, sharing personal LFS-related information tended to occur only after AYAs appraised new partners and the relationship as worth their emotional investment. Similar to findings from research focused on young adults with VHL or HBOC [4, 5, 6], most AYAs reported moments of hesitation, nervousness, or apprehension in anticipation of revealing their LFS diagnosis yet firm beliefs about the importance of sharing the information compelled disclosure. Perceived stigma, the chronicity and uncertainty of LFS, and anticipated negative partner responses contributed to this hesitation and difficult emotions.

Participants reported that disclosing an LFS diagnosis often happened relatively early in romantic partnerships. Communicating this information early fulfilled a sense of moral obligation to respect partners’ reproductive autonomy given the genetic risk for future biological children. Early disclosure also reflected a duty to protect partners’ future well-being as they may enter a committed relationship with someone at high risk of cancer and early mortality due to LFS and who may need substantial support (e.g., informal caregiving). These findings suggest disclosure decisions may be shaped by a broader sense of *genetic responsibility*, a felt duty to determine, manage, and share genetic information for the benefit of others who may be affected by inherited risk [35]. Participants’ disclosure behaviors align with prior work suggesting that genetic knowledge can create obligations not only to family but also to romantic partners and potentially future children [36].

Disclosing LFS toward the beginning of romantic partnerships also served as an emotional self-protection strategy to avoid future rejection or abandonment, which would be profoundly difficult if it occurred in “hard times,” such as during cancer treatment. Other reasons for early disclosure included the onset of LFS-related clinical manifestations (e.g., cancer symptoms) that could not be hidden or that AYAs anticipated would affect the partnership immediately (e.g., dating availability, emotional bandwidth, functional changes).

Participants’ accounts of disclosing LFS to new partners suggest that disclosure of LFS information is a process and that AYAs’ ability to confidently communicate LFS-related information may develop with repeated disclosure enactments. Similar to findings from research focused on young adult survivors of early-onset cancers [9], few AYAs’ reported experiencing negative reactions from partners. However, as relationships progressed, some AYAs with LFS experienced challenges navigating subsequent disclosures regarding challenging topics, such as shortened life expectancy.

Notably, AYAs viewed a partner’s willingness to learn about LFS and try to understand its consequences as a sign of trustworthiness. This relationally responsive behavior appeared to be an important driver of AYAs’ future LFS-related disclosures, depth and breadth of LFS communication, and feelings of closeness and commitment in the relationship (i.e., high relationship quality). This finding is noteworthy given the interrelatedness of relational responsiveness, relationship quality, and mental and physical health [37, 38, 39], the latter of which is often compromised among AYAs with LFS [27, 40].

Our findings corroborate those of past studies regarding the highly interdependent nature of living with LFS among romantic partners [21] and improve our understanding of how this interdependence develops during the formation of relationships among AYAs. AYAs’ narratives suggest that a partner’s responsiveness (e.g., learning LFS) in early phases of the relationship may be a signpost for how the partner and the couple will cope with LFS in a long-term relationship. Young and colleagues found that coping with LFS “in connection” (e.g., mutual support) was associated with more open LFS communication and relational cohesiveness, while coping with LFS independently was linked with more communication challenges and relational discord [21]. To better understand how young couples adapt to LFS over time, future studies could include the perspectives of AYAs’ partners and examine couple coping strategies using dyadic and longitudinal research approaches.

The results of this study also illuminated similarities in disclosure experiences among AYAs with non-LFS heritable cancer syndromes. For example, like AYAs with LFS, young adults with VHL or HBOC syndromes also appraised several aspects of their genetic disease before self-disclosing to dating partners, were apprehensive about disclosure encounters and negative partner responses, and felt concerned about the long-term consequences of genetic disease on the relationship, such as future rejection [4, 5]. Although disclosure message strategies of AYAs with LFS, VHL, and HBOC syndromes are similar, such as directness and transparency regarding risks, differences exist. For example, to avoid rejection, some young adults with VHL described downplaying its impact by emphasizing the word “benign” and avoiding the word “cancer” when self-disclosing to new partners [4]. AYAs with LFS may not have mentioned using such strategies given the high penetrance of malignancies.

### Strengths and limitations

Our ability to examine data from two waves of in-depth interviews enriched this study. However, the study may have been limited by a sample comprised of individuals who were predominantly emerging or young adults (i.e., few adolescents), female, and married or in a long-term relationship. These sample characteristics may have hindered our ability to discern differences in themes by age, sex, or relationship status. We also may have missed examples of negative partner responses, such as rejection, given that nearly 60% of the sample was in a committed long-term relationship. In addition, most AYAs in this study seemed to be skillful communicators of personal LFS information, which may have limited our ability to identify the extent of challenges disclosing LFS to partners. This study also lacked examples of nondisclosure, which has been reported in past research focused on genetic risks and dating [41]. Future research could continue to explore AYAs’ experiences navigating LFS disclosure in new romantic partnerships to confirm or expand on our findings and ensure diverse perspectives are captured.

### Clinical implications

Findings from this study provide clinicians with a foundation for understanding an important quality of life issue among AYAs with LFS: achieving the developmentally normative tasks associated with forming romantic partnerships in the context of non-normative lifelong cancer risk. In developing partnerships, disclosing an LFS diagnosis may be merely the first in a series of LFS-related disclosures. Opportunities to engage in LFS disclosure may increase disclosure efficacy [42]; however, even skillful communicators may need support to feel confident with subsequent disclosures, especially if the topic is emotion- or morally laden, such as disclosing shortened life expectancy, a new cancer diagnosis, or reproductive implications. Concerns about the ability to share accurate LFS information (e.g., genetic inheritance) with new partners can be anxiety-provoking and have major consequences for future relationships (e.g., partner rejection).

Building on findings of past studies focused on non-LFS heritable cancer syndromes [4], AYAs with LFS want and need support to navigate new romantic partnerships as they grow in intimacy. Clinicians (e.g., mental health providers, genetic counselors, physicians, nurses) might use DD-MM concepts and findings from this study to facilitate discussions with AYAs about LFS disclosure strategies and normalize self-disclosure as a challenging process. In therapeutic settings, mental health professionals may work to provide strategies to manage distress and practice disclosure scripts in a safe setting [43, 44]. Clinicians might also provide anticipatory guidance on specific LFS information that could be shared during disclosure to ease fears about partner rejection and address partner worries. Ongoing support from clinicians is critical as AYAs may have varied questions and concerns about new romantic partnerships and LFS-related disclosures as developmentally normative social goals change or AYAs experience prognostic changes. Optimal therapeutic interventions are tailored to the specific needs, apprehensions, and experiences of AYAs, and possibly with their partners. LFS communities might also support AYAs by providing opportunities to discuss experiences, questions, and concerns among peers.

## Conclusions

This study provides a foundation for understanding LFS disclosure in early romantic partnership experiences of AYAs with LFS. In addition to distress associated with the chronic threat of cancer, some AYAs’ social and emotional well-being may be challenged by complicated disclosure decisions and navigating disclosure encounters. Clinicians and LFS communities might provide resources, such as peer support groups and referrals to mental health counseling (as needed), to support AYAs as they navigate these challenges.

## Supplementary Information

Below is the link to the electronic supplementary material.

Supplementary Material 1

## Data Availability

De-identified data will be shared with qualified investigators after IRB review and establishment of appropriate data transfer agreements. Please contact the corresponding author for data requests.
