# Supplementary material for: Navigating disclosure in new romantic partnerships as an adolescent or young adult with Li-Fraumeni syndrome
Source: Fam Cancer. 2025 Sep 10;24(4):71. doi: 10.1007/s10689-025-00495-3 (PMC12423177; doi:10.1007/s10689-025-00495-3)
Supplement: Supplementary file 1 — Supplementary Material 1 [file 10689_2025_495_MOESM1_ESM.docx]

**Appendix 1**

*Interview Questions*

Wave 1

Select questions relevant to the current study:

1. Do you talk with your family about your LFS or the follow-up care that you might need?
2. How open would you say you are with your family members about your experiences with LFS?
3. What topics are you open about (*e.g.*, emotions, cancer effects, the future)? Tell me more about this.
4. How do you tend to communicate about these topics (*e.g.*, verbally, through information seeking/sharing, silently)?
5. What topics are you not open about? Is there anything you specifically avoid?
6. How has talking about LFS-related topics/experiences in your family changed over time, if at all?

Wave 2

Select questions relevant to the current study:

*If currently in a romantic partnership/relationship:*

1. Tell me about your conversations about LFS with your partner. How do you and your partner talk about LFS (*e.g.*, openly, sparingly, only certain topics)?
2. Tell me about your experience of telling your partner about your LFS diagnosis? How did your partner react? How did you feel about their reaction?
3. What is hardest about discussions with your partner? What about your discussions goes well? Is there anything you would say/broach differently?
4. How have your conversations with your partner changed over time? What do you think prompted these changes?
5. How have your interactions about LFS with your partner affected your relationship over time?
6. Are there ways LFS influences what you value in your partner?

*If dating or not currently in a romantic relationship:*

1. Tell me about your conversations about LFS with potential partners, if any.
2. How do you (think you might) talk to potential partners about your LFS diagnosis?
3. What is (what do you think will be) most difficult about discussions with potential partners? (If applicable:) What has gone well?
4. (If applicable:) How have your conversations with potential partners changed over time? What do you think prompted these changes?
5. Are there ways LFS influences what you (will) look for in a potential partner?
